# Detection of latent forms of *Mycobacterium avium* subsp. *paratuberculosis* infection using host biomarker-based ELISAs greatly improves paratuberculosis diagnostic sensitivity

**DOI:** 10.1371/journal.pone.0236336

**Published:** 2020-09-03

**Authors:** Cristina Blanco Vázquez, Marta Alonso-Hearn, Ramón A. Juste, María Canive, Tania Iglesias, Natalia Iglesias, Javier Amado, Fernando Vicente, Ana Balseiro, Rosa Casais

**Affiliations:** 1 Servicio Regional de Investigación y Desarrollo Agroalimentario (SERIDA), Deva Gijón, Asturias, Spain; 2 Animal Health Department, NEIKER-Instituto Vasco de Investigación y Desarrollo Agrario, Derio, Bizkaia, Spain; 3 Servicio Regional de Investigación y Desarrollo Agroalimentario (SERIDA), Villaviciosa, Asturias, Spain; 4 Unidad de Consultoría Estadística, Servicios científico-técnicos, Universidad de Oviedo, Campus de Gijón, Asturias, Spain; 5 Departament of Microbiology and Parasitology, Laboratorio de Sanidad Animal del Principado de Asturias (LSAPA), Gijón, Asturias, Spain; 6 Department of Animal Health, Facultad de Veterinaria, Instituto Ganadería de Montaña (CSIC-ULE), University of León, León, Spain; Universita degli Studi di Sassari, ITALY

## Abstract

Bovine paratuberculosis (PTB) is a chronic granulomatous enteritis, caused by *Mycobacterium avium* subsp. *paratuberculosis* (MAP), responsible for important economic losses in the dairy industry. Current diagnostic methods have low sensitivities for detection of latent forms of MAP infection, defined by focal granulomatous lesions and scarce humoral response or MAP presence. In contrast, patent infections correspond to multifocal and diffuse types of enteritis where there is increased antibody production, and substantial mycobacterial load. Our previous RNA-Seq analysis allowed the selection of five candidate biomarkers overexpressed in peripheral blood of MAP infected Holstein cows with focal (ABCA13 and MMP8) and diffuse (FAM84A, SPARC and DES) lesions vs. control animals with no detectable PTB-associated lesions in intestine and regional lymph nodes. The aim of the current study was to assess the PTB diagnostic potential of commercial ELISAs designed for the specific detection of these biomarkers. The ability of these ELISAs to identify animals with latent and/or patent forms of MAP infection was investigated using serum from naturally infected cattle (n = 88) and non-infected control animals (n = 67). ROC analysis revealed that the ABCA13-based ELISA showed the highest diagnostic accuracy for the detection of infected animals with focal lesions (AUC 0.837, sensitivity 79.25% and specificity 88.06%) and with any type of histological lesion (AUC 0.793, sensitivity 69.41% and specificity 86.57%) improving on the diagnostic performance of the popular IDEXX ELISA and other conventional diagnostic methods. SPARC and MMP8 showed the highest diagnostic accuracy for the detection of animals with multifocal (AUC 0.852) and diffuse lesions (AUC 0.831), respectively. In conclusion, our results suggest that quantification of ABCA13, SPARC and MMP8 by ELISA has the potential for implementation as a diagnostic tool to reliably identify MAP infection, greatly improving early detection of MAP latent infections when antibody responses and fecal shedding are undetectable using conventional diagnostic methods.

## 1. Introduction

Bovine paratuberculosis (PTB) is a chronic granulomatous enteritis caused by *Mycobacterium avium* subsp. *paratuberculosis* (MAP), that is responsible for important economic losses due to reduced milk production, premature culling, reduced slaughter value and continued spread of infection [1, 2]. Furthermore, MAP has a clear zoonotic potential since it has been postulated as a possible trigger factor in several autoimmune diseases in humans such as Crohn’s disease (CD) [3, 4], type I diabetes (T1D) [5], multiple sclerosis (MS) [6, 7] or rheumatoid arthritis (RA) [8, 9]. One mechanism that has been suggested to cause the onset and/or exacerbation of autoimmune disease is molecular mimicry, whereby MAP antigens share sequences or structural similarities with self-antigens [10], so the immune response against MAP antigens could also induced undesirable immune responses against host proteins in genetically predisposed individuals [11].

Two forms of infection, latent or patent, can be distinguished in MAP infected cattle [12]. The disease typically progresses from a latent form with low or moderate frequency of microbiological or humoral immunological evidence of infection, characterized by the presence of focal histological lesions in their intestinal tissues to more severe forms of the disease with a high frequency of microbiological or humoral immunological evidence of infection, in which the granulomatous lesions are patent (multifocal and diffuse lesions readily detected upon microscopic examination of the intestine and associated lymph nodes). A latent form would represent a form of silent PTB that causes no direct losses, but that maintains a hidden MAP reservoir in a herd, while a patent form often corresponds with a visibly clinical disease.

Transmission of MAP primarily occurs by the fecal-oral route through the ingestion of MAP contaminated feces, colostrum, or milk. Infection usually occurs within the first months of life of the animal but remains subclinical for an average of 2–5 years before becoming clinical in a few cases. Spread of PTB is mainly due to its extremely long latent period during which MAP can be shed intermittently into the environment through feces. Thus, early detection and removal of animals in that stage from the herd is critical for PTB control.

Most PTB control programs are based on testing and culling test-positive cows combined with good management practices [13]. Several diagnostic techniques are used to detect MAP infected cattle; however, their performance vary widely depending on the stage of MAP infection [14–16]. Currently available diagnosis methods have low sensitivities and specificities for the detection of latent infection, as the bacteria is excreted in low numbers and animals have low titers of specific antibodies. Fecal culture has been considered the gold standard for diagnosis of MAP but its sensitivity varies from 70% in cattle with PTB-associated clinical signs to 23–29% in infected cattle with no detectable clinical signs [17]. PCR offers a rapid method of assessing MAP status in cultures, feces, tissue and milk [18]. The sensitivity and specificity of fecal PCR were estimated to be 29% and 99.3%, respectively [19]. Recently, Taniguchi et al. [20] have established an association between the amount of MAP DNA in feces, determined by real-time quantitative PCR and the histopathological classification of ileocecal valve lesions. ELISAs, used to detect anti-MAP antibodies, provide rapid results and offer a cheaper alternative to fecal culture and PCR. However, the appearance of an antibody response detectable by ELISA is late as it is associated with disease progression, high shedding, clinical signs and the appearance of severe lesions in gut tissues [16, 21–23]. Thus, the sensitivity of serum specific antibody ELISA varies depending on the stage of infection, being 50–87% in cattle with clinical signs, 24–94% in cattle with no clinical signs but shedding MAP and 7–22% in infected cattle with no clinical signs and no shedding [17]. In general, ELISA specificity varies between 40 and 100% [17] and depends on the particular test, exposure to environmental mycobacteria, concurrent infection with *Mycobacterium bovis*, previous intradermal tuberculosis (TB) test and MAP vaccination. Early stage diagnostics primarily target pro-inflammatory cellular immune responses, the interferon-gamma *(IFN-γ)* release assay (IGRA) uses a sandwich ELISA to detect whether a T-cell mediated immune response has been elicited in response to MAP by measuring the difference in *IFN-γ* signals for whole blood samples activated by a MAP-specific antigenic extract and a non-specific antigen (*M*. *phlei* extract) (ID Vet, Grabels, France) [24]. However, the IGRA only reflects MAP exposure, and thus cannot discriminate between individuals with controlled infection from those with sub-clinical disease, indicating that this method might lead to culling healthy individuals. This means that, even though detection of patent forms can be readily done through different methods, there is not an affordable, sensitive and specific method for detection of latent forms. Therefore, novel diagnostic tools with high sensitivity and specificity are needed to detect latent MAP infections.

The potential of emerging -omic approaches to complement and enhance the diagnosis of MAP infection in cattle has been previously reviewed [23]. Host biomarkers, identified using transcriptomics and proteomics, have been proposed as tools to develop novel diagnostic methods for PTB [25–36]. However, the application of those biomarkers for PTB diagnosis has yet to be developed and properly validated for naturally infected cattle in various stages of infection [37]. Park et al. [38] developed a real-time PCR method based on measuring the gene expression levels of 8 bovine biomarkers (*Timp1*, *Hp*, *Serpine1*, *Tfrc*, *Mmp9*, *Defb1*, *Defb10*, and *S100a8*) in whole blood of cows that might be useful for the diagnosis of PTB disease, including subclinical stage cases.

We previously identified several potential biomarkers for the diagnosis of MAP-infected animals [39]. Whole RNA-sequencing (RNA-Seq) was used to identify host genes differentially expressed in peripheral blood samples collected from animals with focal, or diffuse lesions in gut tissues vs. control animals without detected lesions nor bacterial load in gut tissues. Genes encoding for bovine proteins MMP8 (Matrix metallopeptidase 8) and ABCA13 (ATP binding cassette subfamily A member 13) showed significantly higher expression in animals with focal lesions, while genes encoding for FAM84A (family with sequence similarity 84 member A), SPARC (secreted protein acidic and cysteine rich) and desmin (DES) were up-regulated in animals showing diffuse lesions when compared with the control animals (Table 1). In the present study, these five proteins were selected for their validation as biomarkers for the different PTB forms of infection by ELISA according to their high expression levels in peripheral blood, their cellular location (extracellular proteins, present in sera) and commercial availability of ELISA kits for its specific detection.

**Table 1:** Differential expression of host biomarker genes selected after RNA-Seq analysis of peripheral blood of Holstein cattle with focal and diffuse histological lesions vs. control animals without detected lesions.

Biomarker ID	Gene ID	Focal vs. Control		Diffuse vs. Control	
		Mean Log2 fold change	P-value	Mean log2 fold change	P-value
MMP8	XLOC_010350	1.89	0.00035		
ABCA13	XLOC_033714	3.74	0.00005		
FAM84A	XLOC_005071			2.15	0.00005
SPARC	XLOC_040915			2.21	0.00065
DES	XLOC_019473			3.75	0.00030

The mean Log2 fold change, is a measurement (ratio) of differential gene expression between two compared groups expressed as a logarithm; MMP8, bovine matrix metallopeptidase 8; ABCA13, bovine ATP binding cassette subfamily A member 13; FAM84A, bovine family with sequence similarity 84 member A; SPARC, bovine secreted protein acidic and cysteine rich; DES. bovine desmin.

The aim of this study was to evaluate the diagnostic potential of commercial ELISAs based on detection of these five bovine biomarkers to detect latent and patent forms of MAP infection in naturally infected cattle, using reference serum samples from well characterized animals with focal, multifocal and diffuse histological lesions in their intestinal or associated lymphoid tissues [40].

## 2. Materials and methods

### 2.1 Animals, samples and ethical considerations

Two groups of animals (n = 155) were included in this field study: **Slaughtered group)** Ninety-four Holstein Friesian cows (ranging from 0.81 to 12.66 years of age) came from 26 farms located in the Principality of Asturias (Northwest of Spain). In Asturias, 32.6% of the herds and 1.9% of the animals were positive by serum ELISA in 2019 (unpublished data from the Regional Government). Specifically, fifty-six animals came from a dairy farm with a mean herd size of 105 cows (2016–2019) and a mean prevalence of PTB of 6.30% in the sampling period, based on serum ELISA test (IDEXX laboratories, Hoofddorp, The Netherlands). The infection status of these 56 animals was also determined once a year by fecal culture and real time PCR (2016–2019). Another thirty-four animals were randomly selected from cows slaughtered in a local abattoir (coming from 24 different farms) and four more cows were culled from the SERIDA's Friesian cow farm (3% mean prevalence of PTB in the period 2018–2019). The PTB infection status of these 94 animals at the time of slaughter was determined by histopathology, specific antibody serum ELISA test, and bacteriological culture and specific real-time PCR of tissues and feces; **and PTB-free group)** Sixty-one animals (ranging from 0.50 to 10.08 years of age) came from a farm in Asturias with 0% prevalence of PTB. The PTB-free status of this farm was verified yearly by repeatedly negative IDEXX serum ELISA results and lack of a history of clinical PTB in the period 2016–2019, as well as by bacteriological culture and specific fecal real-time PCR in 2019.

Samples of serum and feces included in this field study were collected from all animals while tissue samples were only taken from the slaughtered animals *in situ* at the local abattoir after evisceration and split for microbiological and histopathological processing.

Experimental procedures were approved by the SERIDA Animal Ethics Committee and authorised by the Regional Ministry of Agro-livestocks and Indigenous Resources of the Principality of Asturias (authorization codes PROAE 29/2015 and PROAE 66/2019). All the procedures were carried out in accordance with Directive 2012/63/EU of the European Parliament (Guidelines for the Care and Use of Animals for Research Purposes). Peripheral blood and fecal samples were collected by trained personnel and in accordance with good veterinary practice.

### 2.2 Histopathological classification of animals

Tissue sections of distal jejunum, ileocecal valve (ICV) and, jejunal and ileal lymph nodes were collected from the 94 slaughtered cows, fixed in 10% neutral buffered formalin, sliced and embedded in paraffin wax using standard procedures. Afterwards, 4 μm sections were assessed by haematoxylin-eosin (HE) and Ziehl-Neelsen (ZN) staining for specific acid-fast bacteria (AFB) detection. Slices were observed using an Olympus BH-2 light microscope (Olympus, Tokyo, Japan) and photographed using an Olympus DP-12 digital camera (Olympus, Tokyo, Japan). The stained sections were examined by light microscopy for AFB and evidence of MAP disease-specific pathological lesions, and were classified in four groups: focal, multifocal, diffuse and with no detectable PTB-associated lesions [40]. Briefly, the focal lesions consist of granulomas, mainly located in the jejunal and ileal lymph nodes, and not affecting the intestinal lamina propia. The multifocal lesions consist of well-demarcated granulomas in the intestinal lymphoid tissue and also in the intestinal lamina propia. The diffuse lesions were characterized by severe and diffuse granulomatous enteritis and lymphadenitis, which markedly altered the normal histological structure. According to the inflammatory cell type present in the infiltrate and the amount of acid-fast bacilli (AFB), diffuse lesions were subdivided into diffuse lymphoplasmocytic or paucibacillary, diffuse intermediate and diffuse histiocytic or multibacillary lesions [41]. The focal, multifocal, diffuse and with no detectable PTB-associated lesions groups correspond to the previously described latent (focal), patent (multifocal and diffuse) and apparently free (no lesions) forms of infection [42].

### 2.3 Enzyme-linked immunosorbent assay (ELISA) for the detection of MAP-specific antibodies

Blood (8 mL) was collected from the coccygeal vein of each animal into 4.5 mL serum clot activator Vacutainer® tubes (Vacuette, Kremsmunster, Austria). Serum was separated after clotting by centrifugation (2500 x g for 20 min) and stored at 20°C until use. Serum samples were tested using the *Mycobacterium paratuberculosis* Antibody test kit (IDEXX laboratories, Oofddorp, The Netherlands) according to the manufacturer´s instructions. The optical density (OD) in each well was measured at 450nm by an ELISA plate reader model 680 (SIGMA, St. Lois, MO, USA). The measured ODs were normalized and the results were expressed as a percentage of the positive control OD according to the following formula: % relative OD sample/ OD positive control = 100 x [(ODsample_+Ag_ -ODsample_-Ag_) / (OD mean positive control_+Ag_−OD mean positive control_-Ag_)].

### 2.4 Bacteriological culture of tissues and feces

For bacteriological culture, a pool (2 gr) of ileocecal lymph nodes, distal jejunal lymph node, ICV, and distal jejunum were decontaminated with 38 mL of hexa-decyl pyridinium chloride at a final concentration of 0.75% (Sigma, St. Louis, MO, USA) and homogenized in a Stomacher blender. After 30 min of incubation at room temperature, a 15 mL aliquot of the suspension was transferred to a new tube and left overnight for decontamination and sedimentation. Approximately, 200 μl of the suspension was taken from the layer right over the sediment and inoculated into two slants of Herrolds egg yolk medium (HEYM; Becton Dickinson, Sparks, MD, USA) and into two slants of Lowenstein-Jensen medium (LJ; Difco, Detroit, MI, USA), both supplemented with 2mg/L of Mycobactin J (ID.vet Innovative Diagnostics, Grabels, France). Feces were taken from the rectum of each animal, maintained at 4°C and processed within 48 h after arrival at the laboratory. The fecal samples (2 g each) were decontaminated, blended in a Stomacher, and cultured in HEYM and LJ, as previously described for tissue culture.

### 2.5 Tissues and fecal real-time polymerase chain reaction (PCR)

Isolation of genomic DNA from tissues and feces was performed using the MagMax Total Nucleic Acid Isolation kit according to the manufacturer’s instructions (TermoFisher Scientifc, Lissieu, France). For detection of MAP DNA, the LSI VetMax Triplex real-time PCR was used according to the manufacturer’s instructions (TermoFisher Scientifc, Lissieu, France). The kit enables real-time PCR detection of *Map* IS900 and F57 genes in DNA extracted from feces, liquid cultures, and tissues or colonies. Real-time PCR amplifications were performed using the MX3000P Real-Time PCR detection system (Stratagene, San Diego, USA) with the following conditions: 1 cycle at 50°C for 2 min, 1 cycle of 95°C for 10 min, 45 cycles of denaturation at 95°C for 15 s, and annealing/extension at 60°C for 60 s.

### 2.6 ELISA for biomarkers detection

The concentration of the selected biomarkers in the serum of each animal were measured using commercially available ELISAs according to the manufacturers´ instructions (MyBioSource, San Diego, CA. USA). Quantitative sandwich ELISA kits Bovine Matrix Metalloproteinase 8 (Detection range 3.12–100 ng/mL); Bovine Protein FAM84A ELISA kit (Detection range 62.5–2000 pg/mL), Bovine SPARC ELISA kit (Detection range 0.78–50 ng/mL), and competitive Bovine ATP-binding cassette sub-family A member 13 ELISA kit (Detection range 1–5000 pg/mL) and Bovine Desmin ELISA kit (Detection range 0–25 ng/mL) were used for specific detection of MMP8, FAM84A, ABCA13, SPARC and DES, respectively. A standard curve was used to determine the concentration of each biomarker in the serum samples (average OD of each standard was plotted on the vertical axis against the concentration on the horizontal axis and the best fit drawn to generate a regression curve). Standards and samples were tested in duplicate. The mean value of the blank control was subtracted from mean raw OD values before result interpretation. The concentration of the biomarkers in each sample was interpolated from the standard curve. For optimization various dilutions of the serum were tested (for instance: undiluted, 1:2, 1:4 and 1:8) and the dilution which showed a larger number of samples with measurement values included within the range of the standard curve was considered optimal. Regarding the repeatability and reproducibility of these ELISAs the supplier indicates that both the intra-plate and inter-plate variability expressed as coefficient of variation (CV% = standard deviation/mean of replicates x 100) for FAM84A and MMP8 are less than 15%, the intra-plate CV is ≤6.6% and the inter-plate CV≤11.3% for SPARC and both the intra-lot and inter-lot CV are less than 10% for DES and ABCA13.

### 2.7 Statistical analysis

Data obtained from biomarkers quantification was analyzed using the pROC, OptimalCutpoints and Caret packages of R program Statistical environment version 3.6.0 (http://www.R-project.org/), with confidence intervals stated at 95% for the final results.

The AUC (area under the curve) and optimal cut off value for each biomarker-based ELISA was determined individually by Receiver operator characteristic (ROC) curve analysis. The optimal cut off values for sensitivity and specificity were based on maximum Youden Index (J = Se+Sp-1). The discriminatory power of each biomarker to discern between the different histopathological groups and the control group was determined as follows: biomarker-based ELISAs with AUC values ≥0.9 were considered to have excellent discriminatory power; 0.8≤AUC<0.9 good discriminatory power; 0.7≤AUC <0.8 fair discriminatory power; and AUC <0.7, poor discriminatory [38, 43].

Multivariate binary logistic regression models (Caret package of R) were used to assess the diagnostic capacity of the simultaneous use of several biomarkers providing AUC, sensitivity and specificity values for the different biomarker combinations.

Comparison of ROC curves to test the statistical significance of the difference between the areas under ROC curves (derived from the same cases) was performed for the biomarkers with fair, good and excellent AUC values (AUC≥0.7) within each histopathological group using the DeLong method [44].

The proc FREQ of the SAS statistical package (SAS Inc., Cary, NC, USA) was used for agreement analysis between pairs of diagnostic assays. The coefficient of agreement (kappa (κ)) was interpreted as follows: κ = 0.00–0.20, poor; κ = 0.21–0.40, fair; κ = 0.41–0.60, moderate; κ = 0.61–0.80, good; and κ = 0.81–1.00, excellent agreement.

## 3. Results

### 3.1 Histopathological, immunological and microbiological assessment of MAP infection status

The histopathological, immunological and microbiological characteristics of the animals included in this study, slaughtered animals (n = 94) and control animals from a PTB-free farm (n = 61), are summarized in Table 2. The complete dataset is available in S1 Table. Pathological examination of intestinal tissue sections allowed the classification of the animals in four groups according to the type and extension of the histopathological lesion: focal (n = 55, 58.51%), multifocal (n = 18, 19.14%), diffuse (n = 15, 15.95%) and with no detectable histological lesions (n = 6, 6.38%).

**Table 2:** Assessment of MAP infection status in 155 Holstein Friesian cows included in the study.

Diagnostic method	Focal (n = 55)	Multifocal (n = 18)	Diffuse (n = 15)	NL (n = 6)	PTB-free farm (n = 61)
ZN	49.09%	94.44%	100.00%	0%	UN
IDEXX ELISA	5.45%	27.78%	73.33%	0%	0%
FECAL PCR	9.09%	33.33%	73.33%	0%	0%
FECAL CULTURE	5.45%	16.67%	26.66%	0%	0%
TISSUE PCR	28.30%	44.44%	73.33%	0%	UN
TISSUE CULTURE	28.30%	44.44%	66.66%	0%	UN
CLINICAL SIGNS	0.00%	21.43%	64.28%	0%	0.00%

NL, refers to the 6 control animals with no PTB-associated lesions detected in their gut tissue; PTB-free farm; refers to the 61 control animals from a PTB-free farm; UN, undetermined (tissues not available as animals were alive); The calculation of the % of animals with clinical signs is based on animals with known clinical status (focal n = 28, multifocal n = 14, diffuse n = 14, NL n = 4 and PTB-free farm n = 61).

In the group of animals with focal lesions, 76.36% were positive by one or more diagnostic methods (ZN, fecal and tissue real-time PCR, fecal or tissue bacteriological culture or serum ELISA). Specifically, 49.09% were positive by ZN, 5.45% by both fecal bacteriological culture (low bacterial load, <10 CFU/gr), and serum ELISA, 9.09% by fecal real-time PCR, and 28.30% by both tissue real-time PCR and by tissue bacteriological culture. None of the animals in the focal group (0 out of 28 animals with known clinical status) showed clinical signs associated with PTB.

In the group of animals with multifocal lesions, 100% of the animals were positive for at least one of the following 6 techniques: ZN, fecal and tissue real-time PCR, fecal and tissue bacteriological culture and serum ELISA. Specifically, 94.44% of the animals were positive by ZN, 27.78% by serum ELISA, 33.33% by fecal real-time PCR, 44.44% by both tissue real-time PCR and tissue culture and 16.67% by fecal culture (one animal with low and two with heavy bacterial load). In this group 21.43% of the animals (3 out of 14 with known clinical status) showed clinical signs.

In the diffuse group, 100% of the animals were positive by ZN. Serum ELISA and both fecal and tissue real-time PCR analysis showed, in each case 73.33% positives. Fecal and tissue culture analysis revealed 26.66% and 66.67% positives, respectively, both with heavy bacterial loads (>50 CFU/gr). In this group, 64.28% (9 out of 14 with known clinical status) of the animals had PTB-associated clinical signs.

Only 6 out of the 94 animals analyzed by histopathology did not show any detectable histological lesion in their intestinal tissues and associated lymph nodes. These 6 animals were negative by fecal and tissue MAP-specific PCR and bacteriological culture, and by serum ELISA in the period 2016–2019. Animals in the PTB-free group were negative by IDEXX serum ELISA (2016–2019), and negative by bacteriological culture and specific fecal real-time PCR in 2019. No animals in this group showed clinical signs in the period 2016–2019. Given the difficulty in finding negative animals without lesions it was decided to establish a non-infected control group consisting of the 6 animals without PTB-associated detected lesions and the 61 live animals from the PTB-free farm. This served to increase the sample size and improve the statistical significance of the results.

### 3.2 ROC analysis of the selected biomarker-based ELISAs

The diagnostic accuracies of each biomarker-based ELISA to discriminate between the different histopathological groups and the control group was investigated by ROC analysis (Table 3). The AUCs, sensitivities, and specificities values were estimated for each biomarker individually and in combination based on optimal cut off values. The diagnostic performance of the biomarker-based ELISAs was also compared to that of the specific anti-MAP antibody ELISA (IDEXX ELISA). ROC analysis of the ELISAs for the detection of 4 biomarkers (FAM84A, DES, MMP8 and SPACR) and the IDEXX ELISA was performed using 88 serum samples from 55 animals with focal, 18 with multifocal and 15 with diffuse lesions while ROC analysis of the ABCA13- based ELISA was performed using 85 serum samples from 53 animals with focal, 17 with multifocal and 15 with diffuse lesions.

**Table 3:** Diagnostic performance of biomarker-based ELISAs for diagnosis of cattle with different types of PTB-associated histopathological lesions in their intestinal tissue.

ELISA	AUC	P value	CUT OFF	SE (%)	SP (%)	DV	Kappa (SER)
**FOCAL (N = 55) VS. CONTROL (N = 67)**							
FAM84A	0.506	0.906	1.23	30.91	77.61	0.543	0.0885 (0.0836)
DES	0.625	0.018	23.1	61.82	68.66	0.652	0.3047 (0.0866)
**ABCA13**^**a1**^	**0.837**	**<0.001**	**1.74**	**79.25**	**88.06**	**0.837**	**0.6771 (0.0678)**
MMP8	0.771	<0.001	33.31	87.27	64.18	0.757	0.5007 (0.0747)
SPARC	0.802	<0.001	273.15	59.26	86.57	0.729	0.4692 (0.0797)
IDEXX^b^	0.541	0.434	28.39	14.55	100.00	0.573	0.1575 (0.0519)
IDEXX^c^			55.00	5.45	100.00	0.527	0.0596 (0.0336)
**MULTIFOCAL (N = 18) VS. CONTROL (N = 67)**							
FAM84A	0.507	0.927	0.87	50.00	64.18	0.571	0.1087 (0.1018)
DES	0.598	0.206	23.93	61.11	68.66	0.649	0.2318 (0.1035)
ABCA13^a2^	0.642	0.073	0.97	70.59	65.67	0.681	0.2599 (0.0973)
MMP8	0.739	0.002	26.92	94.44	58.21	0.763	0.3400 (0.0787)
**SPARC**	**0.852**	**<0.001**	**354.94**	**66.67**	**92.54**	**0.796**	**0.6043 (0.1078)**
IDEXX^b^	0.600	0.197	67.58	27.78	100.00	0.639	0.3775 (0.1244)
IDEXX^c^			55.00	27.78	100.00	0.639	0.3775 (0.1244)
**DIFFUSE (N = 15) VS. CONTROL (N = 67)**							
FAM84A	0.616	0.162	1.06	66.67	71.64	0.692	0.2812 (0.1058)
DES	0.621	0.147	36.59	46.67	88.06	0.674	0.3473 (0.1299)
ABCA13^a3^	0.81	<0.001	1.20	86.67	77.61	0.821	0.4810 (0.1011)
**MMP8**	**0.831**	**<0.001**	**40.23**	**100.00**	**71.64**	**0.858**	**0.4803 (0.0893)**
SPARC	0.599	0.235	7.73	100.00	32.84	0.664	0.1517 (0.0450)
**IDEXX**^**b**^	**0.918**	**<0.001**	**19.60**	**86.67**	**97.01**	**0.918**	**0.8368 (0.0791)**
IDEXX^c^			55.00	73.33	100.00	0.867	0.8180 (0.0873)
**MULTIFOCAL + DIFFUSE (n = 33) VS. CONTROL (N = 67)**							
FAM84A	0.557	0.358	1.06	51.52	71.64	0.616	0.2263 (0.1005)
DES	0.608	0.08	22.88	63.64	67.16	0.654	0.2860 (0.0955)
ABCA13^a4^	0.721	<0.001	1.17	68.75	76.12	0.724	0.4277 (0.0938)
**MMP8**	**0.781**	**<0.001**	**26.92**	**96.97**	**58.21**	**0.776**	**0.4569 (0.0735)**
SPARC	0.737	<0.001	40.61	96.97	38.81	0.679	0.2736 (0.0624)
IDEXX^b^	0.745	<0.001	19.60	54.55	97.01	0.758	0.5729 (0.0883)
IDEXX^c^			55.00	48.48	100.00	0.734	0.5579 (0.0876)
**ALL LESIONS (N = 88) VS. CONTROL (N = 67)**							
FAM84A	0.525	0.590	1.06	42.05	71.64	0.568	0.1295 (0.0724)
DES	0.618	0.012	23.10	61.36	68.66	0.650	0.2939 (0.0756)
**ABCA13**^**a5**^	**0.793**	**<0.001**	**1.39**	**69.41**	**86.57**	0.780	**0.5437 (0.0664)**
MMP8	0.775	<0.001	33.17	87.50	64.18	0.758	0.5290 (0.0686)
SPARC	0.777	<0.001	291.55	51.72	88.06	0.699	0.3706 (0.0626)
IDEXX^b^	0.618	0.012	28.39	28.41	100.00	0.642	0.2554 (0.0482)
IDEXX^c^			55.00	21.59	100.00	0.608	0.1923 (0.0427)

AUC, area under the curve; P-value, it is the p-value of the AUC area, indicates whether the discrimination between animals with focal, multifocal, diffuse or any type of lesions and controls is significant; The cut-off point is expressed as ng/mL for the biomarkers and as a % of the positive reference sample for the IDEXX ELISA; SE, sensitivity; SP, specificity; DV, diagnostic value (semi-sum of the sensitivity and specificity); Kappa, coefficients of agreement; SER, standard error; VS., versus; The control group consists of 67 animals, 6 animals with no lesions detected and 61 from a PTB-free farm; DES, bovine desmin; FAM84A, bovine family with sequence similarity 84 member A; ABCA13, bovine ATP binding cassette subfamily A member 13; MMP8, bovine matrix metallopeptidase 8; SPARC, bovine secreted protein acidic and cysteine rich; a1, a2, a3, a4 and a5 indicate that the number of animals with focal, multifocal, diffuse or with any type of lesions analysed for ABCA13 was 53, 17, 15 and 85, respectively; b, the diagnostic accuracy of the IDEXX ELISA was calculated by ROC curve analysis; c, the cut off value used to calculate the sensitivity and specificity of the assay was calculated using the cut off value established by the supplier. The ELISAs with the best diagnostic performance are shown in bold face.

**Fig 1:**
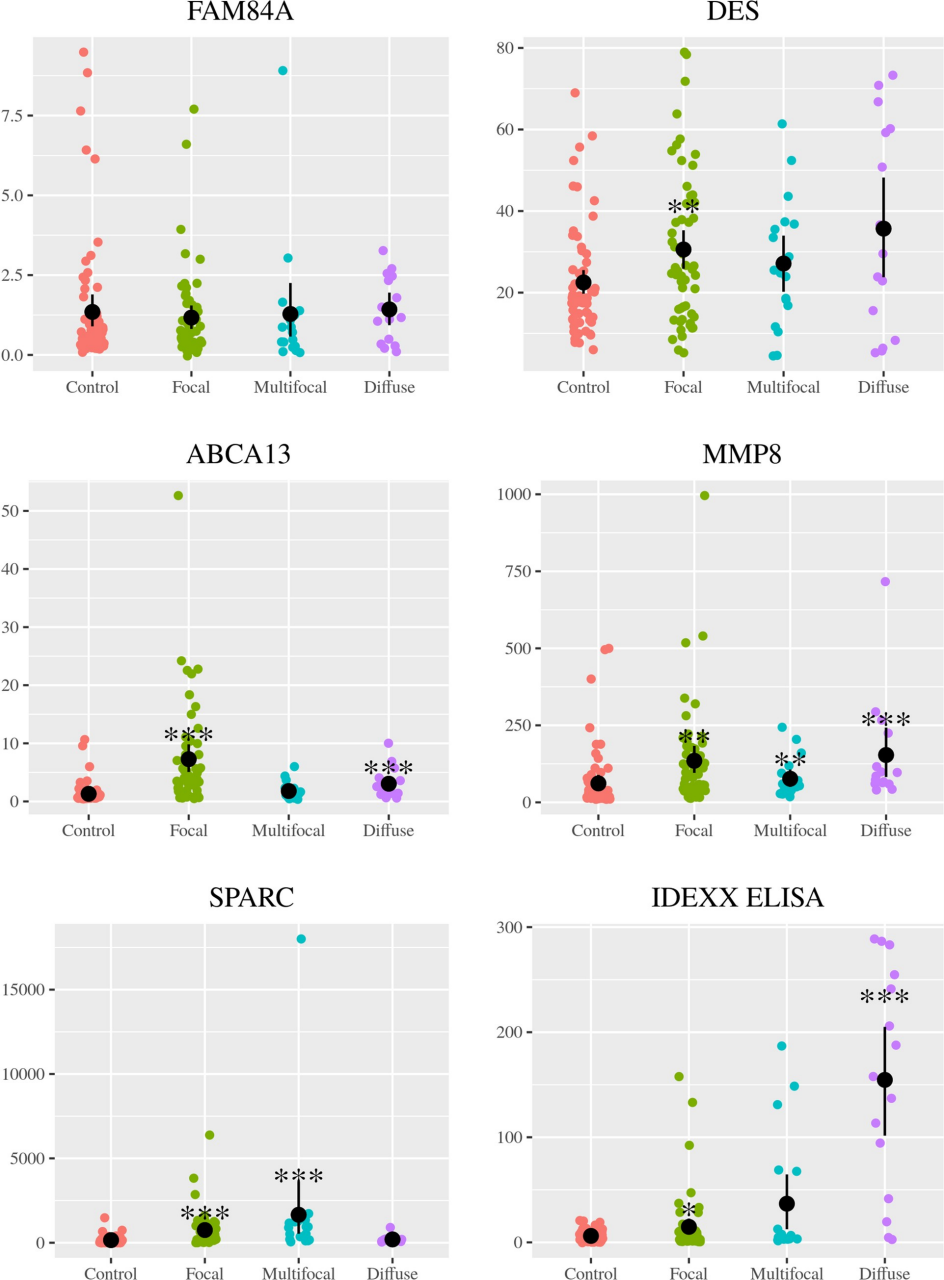
Biomarker expression levels in serum of Holstein Friesian cattle showing different types of histological lesions consistent with PTB in their intestinal tissues (focal (n = 55), multifocal (n = 18), and diffuse (n = 15)) and in non-infected control animals (n = 67). The number of animals with focal, multifocal, diffuse or with any type of lesions represented in the plot for the ABCA13-based ELISA was 53, 16, 14 and 83, respectively. Biomarkers were detected and quantified in bovine serum by specific ELISAs supplied by MyBioSource, San Diego, CA, USA. FAM84A, bovine family with sequence similarity 84 member A; DES, bovine desmin; ABCA13, bovine ATP binding cassette subfamily A member 13; MMP8, bovine matrix metallopeptidase 8; SPARC, bovine secreted protein acidic and cysteine rich; IDEXX ELISA. The data are represented as scatter plots with each dot representing a single animal. The mean of each histopathological group is represented by a gross black point and the standard deviation by a vertical line. The asterisks indicate if the differences between each histopathological group and the control are or not significant (*, p<0.05; **, p<0.01; ***, p<0.001).

**Fig 2:**
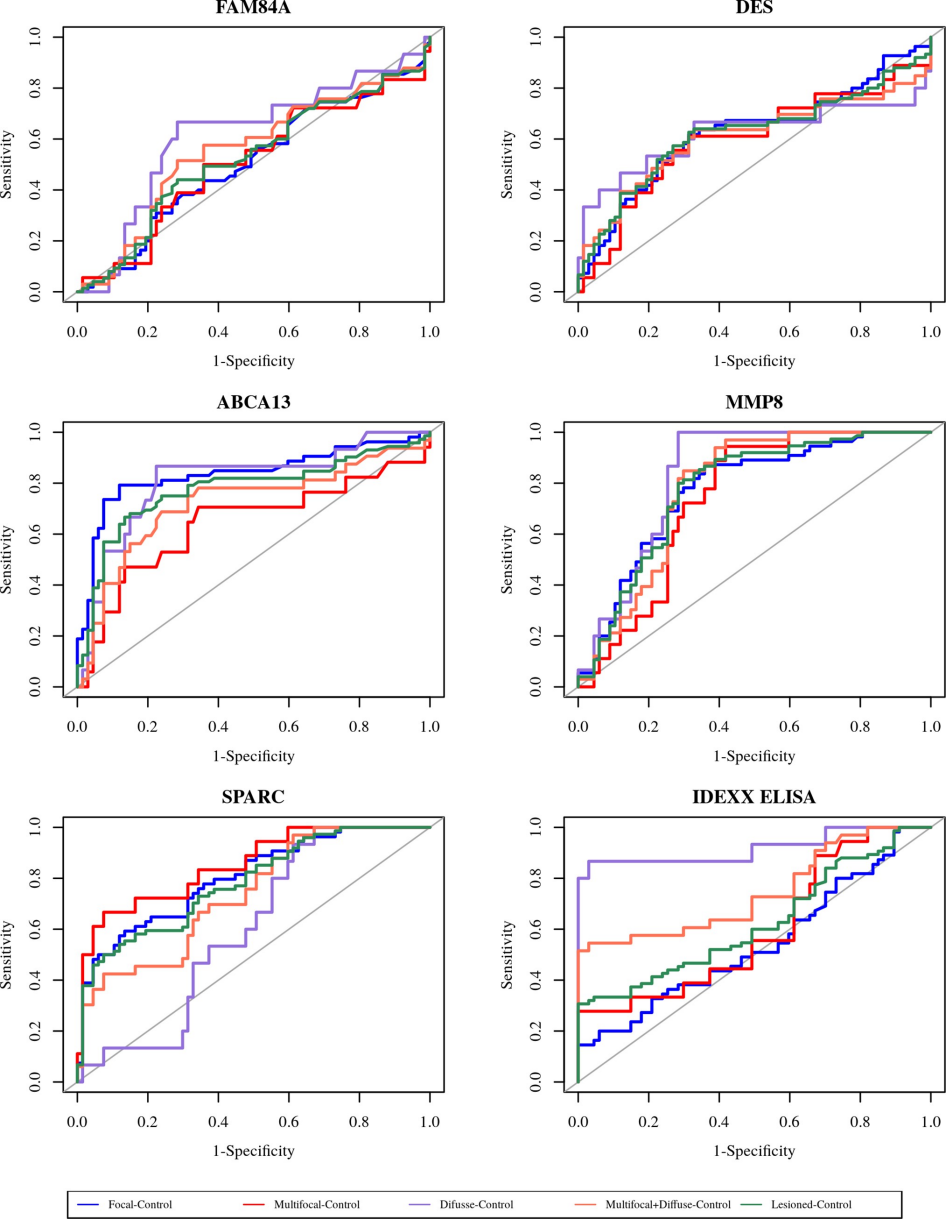
Receiver operator characteristic curves (ROC curves) of selected biomarkers in Holstein Friesian cows with focal (n = 55), multifocal (n = 18) or diffuse (n = 15) histological lesions in their intestinal tissues vs. non-infected control animals (n = 67). The number of animals with focal, multifocal, diffuse or with any type of lesions represented for ABCA13 was 53, 17, 14 and 84, respectively. FAM84A, bovine family with sequence similarity 84 member A; DES, bovine desmin; ABCA13, bovine ATP binding cassette subfamily A member 13; MMP8, bovine matrix metallopeptidase 8; SPARC, bovine secreted protein acidic and cysteine rich; IDEXX, IDEXX serum ELISA.

The expression levels of each biomarker in the serum of every single Holstein Friesian cattle within the different histopathological groups (focal, multifocal, and diffuse) and in the non-infected control group are shown in Fig 1. ROC curves of each selected biomarker for the different histopathological groups when compared to the non-infected control group are shown in Fig 2.

#### 3.2.1 Detection of animals with focal histological lesions

Three biomarkers had fair (MMP8, 0.7≤AUC<0.8) or good (ABCA13 and SPARC, 0.8≤AUC<0.9) discriminatory power between the focal and control groups, while the rest of the biomarkers had poor discriminatory power (AUC<0.7). The ABCA13-based ELISA showed the most accurate diagnostic performance with an AUC value of 0.837 (95% confidence interval [CI]: 0.757–0.917, p<0.001), a sensitivity of 79.25% and a specificity of 88.06%. Comparison of the ROC curves of biomarker-based ELISAs with AUCs≥0.7 (ABCA13, MMP8 and SPARC) showed that there were no significant differences between the three ROC curves (p>0.05 in all cases). We also tested whether using a combination of biomarker-based ELISAs increases sensitivity of the method. Logistic regression analysis indicated that biomarkers FAM84A and MMP8 were excluded and DES, ABCA13 and SPARC were included in the diagnostic model for discrimination of animals with focal lesions and control animals. The AUC value of this diagnostic model reached 0.911 (95% CI: 0.859–0.964), and the model exhibited 84.62% sensitivity and 86.57% specificity. Diagnostic performance of ABCA13, MMP8 and SPARC for the detection of animals with focal lesions was better than that of the IDEXX ELISA which had an AUC value of 0.541 (95% CI: 0.437–0.646, p>0.05), a sensitivity of 14.55% and a specificity of 100.00%.

#### 3.2.2 Detection of animals with multifocal histological lesions

Two biomarkers had fair (MMP8, 0.7≤AUC<0.8) or good (SPARC, 0.8≤AUC<0.9) discriminatory power between the multifocal and control groups, while the rest of the biomarkers had poor discriminatory power. The SPARC-based ELISA showed the most accurate diagnostic performance with an AUC value of 0.852 (95% CI: 0.749–0.954, p<0.001), a sensitivity of 66.67% and a specificity of 92.54%. Comparison of the ROC curves of MMP8 and SPARC-based ELISAs (AUCs≥0.7) showed that there were no significant differences between their ROC curves (MMP8 vs. SPARC, p = 0.154). Logistic regression analysis indicated that ABCA13 and MMP8 were excluded and biomarkers FAM84A, DES and SPARC were included in the diagnostic model for discrimination of animals with multifocal lesions and control animals. The AUC of the diagnostic model was 0.851 (95% CI: 0.737–0.964) with a 70.54% sensitivity and 89.55% specificity. Diagnostic performance of MMP8 and SPARC-based ELISAs for detection of animals with multifocal lesions was better than that of the IDEXX ELISA which had an AUC value of 0.600 (95% CI: 0.446–0.754, p>0.05), a sensitivity of 27.78% and a specificity of 100.00%.

#### 3.2.3 Detection of animals with diffuse histological lesions

ABCA13 and MMP8-based ELISAs had good discriminatory power between the diffuse and control groups (0.8≤AUC<0.9), while the rest of the biomarkers had poor discriminatory power (AUC<0.7). The MMP8-based ELISA showed the most accurate diagnostic performance with an AUC value of 0.831 ((0.744–0.917, 95% CI), p<0.001) a sensitivity of 100.00% and a specificity of 71.64%. The diagnostic performance of the IDEXX ELISA for the detection of animals with diffuse lesions was better than that of the biomarker-based ELISAs with an AUC value of 0.918 (95% CI: 0.810–1.000, p<0.001), a sensitivity of 86.67% and a specificity of 97.01%. Comparison of the ROC curves of ELISAs with AUCs≥0.7 (ABCA13, MMP8 and IDEXX ELISA) showed that there were no significant differences between the three ROC curves (p>0.05 in all cases). Logistic regression analysis did not find any significant diagnostic model for the discrimination of animals with diffuse lesions and control animals.

#### 3.2.4 Detection of animals with multifocal and diffuse histological lesions

ABCA13, MMP8 and SPARC-based ELISAs had fair (0.7≤AUC<0.8) discriminatory power between animals with multifocal and diffuse lesions and the non-infected control group. The MMP8-based ELISA showed the most accurate diagnostic performance with an AUC value of 0.781 (95% CI: 0.692–0.869, p<0.001) a sensitivity of 96.97% and a specificity of 58.21%. Diagnostic performance of the MMP8-based ELISA for detection of animals with multifocal and diffuse lesions was better than that of the IDEXX ELISA which had an AUC value of 0.745 (95% CI: 0.632–0.858, p<0.001), a sensitivity of 54.55% and a specificity of 97.01%. Comparison of the ROC curves of ELISAs with AUCs≥0.7 (ABCA13, MMP8, SPARC and IDEXX) showed that there were no significant differences between the four ROC curves (p>0.05 in all cases). Logistic regression analysis indicated that biomarkers DES, ABCA13, MMP8 and SPARC-based ELISAs can be included in a diagnostic model for discrimination of animals with multifocal and diffuse lesions and the control animals. The AUC value of the diagnostic model reached 0.820 (95% CI: 0.727–0.910), and the model had 75% sensitivity (75%) and 85.07% specificity.

#### 3.2.5 Detection of animals with focal, multifocal and diffuse histological lesions

For the detection of animals with any type of histological lesions (focal, multifocal or diffuse) we compared these animals with the control group. It must be taken into account that in this analysis the three different histopathological groups are not equally represented (focal n = 55, multifocal n = 18 and diffuse n = 15), however, this is a reflection of the real situation on farms [42]. ABCA13, MMP8 and SPARC-based ELISAs had fair (0.7≤AUC<0.8) discriminatory power between animals with any type of lesion and the control group. The ABCA13-based ELISA showed the most accurate diagnostic performance with an AUC value of 0.793 (95% CI: 0.719–0.867, p<0.001), a sensitivity of 69.41% and a specificity of 86.57%. However, comparison of the ROC curves of biomarker-based ELISAs with AUCs≥0.7 (ABCA13, MMP8 and SPARC) showed that there were no significant differences between the three ROC curves (p>0.05 in all cases). Logistic regression analysis indicated that biomarker FAM84A was excluded and biomarkers DES, ABCA13, MMP8 and SPARC-based ELISAs included in the diagnostic model for discrimination of infected animals and non-infected control animals. The AUC value of the model reached 0.878 (95% CI: 0.824–0.932) with an 80.95% sensitivity and 85.07% specificity. The diagnostic performance of the ABCA13, MMP8 and SPARC-based ELISAs was better than that of the IDEXX ELISA which had an AUC value of 0.618 (95%CI: 0.53–0.705, p<0.012), a sensitivity of 28.41% and a specificity of 100.00%.

### 3.3 Correlation between the histopathological classification of the lesions and the ELISA results

The coefficients of agreement (κ values) between the histopathological classification of lesions and the results of the ELISAs are also included in Table 3. The best coefficients of agreement between the focal, multifocal and diffuse histopathological groups and the biomarker-based ELISA results were obtained for biomarker ABCA13 (good agreement, κ = 0.6771), SPARC (good agreement, κ = 0.6043) and MMP8-based ELISA (moderate agreement, κ = 0.4803), respectively. The agreement between the histopathological findings and the biomarker-based ELISA results was worse when the multifocal and diffuse lesion groups were grouped. In this case, the best result was obtained for the MMP8-based ELISA which showed a moderate agreement (κ = 0.4569). The best coefficient of agreement between the group of animals with any type of lesion and the biomarker-based ELISA results was obtained for ABCA13-based ELISA with a moderate κ value of 0.5437. An excellent agreement (κ = 0.8368) was obtained between the histopathological classification of animals with diffuse lesions and the IDEXX ELISA results. In the rest of the histopathological groups, the coefficient of agreement of the biomarker-based ELISAs was better than that of the IDEXX ELISA. The agreement coefficients were consistent with the AUC values obtained by ROC analysis.

### 3.4 Comparison of the diagnostic performance of bovine biomarker-based ELISAs with that of conventional methods for the detection of animals with focal lesions and any type of lesion

The diagnostic performance of the ABCA13, MMP8 and SPARC-based ELISAs for the focal and any type of lesion groups was compared with the IDEXX ELISA and additionally with other conventional PTB diagnosis methods such as specific fecal and tissue real-time PCR and bacteriological culture (Table 4). The biomarker-based ELISAs showed a better diagnostic value than the other diagnostic methods tested for the detection and discrimination of animals with focal lesions. For instance, the ABCA13-based ELISA detected as positive 42 out of 53 animals with focal lesions (79.25% sensitivity) and as negative 59 out of the 67 controls (88.06% specificity). Specifically, the IDEXX ELISA was 100% specific but only detected 3 out of 55 animals with focal lesions (5.45% sensitivity) when the cut off used was the one established by the supplier (55%) or 8 out of 55 (14.54%) when the cut-off point established by ROC analysis was used (28.39%). Ante-mortem assays like fecal culture and PCR were 100% specific but only detected 3 out of 55 (5.45%) and 5 out of 55 (9.09%) animals with focal lesions, respectively. Post-mortem assays like tissue culture and PCR were 100% specific and detected a higher percentage of animals with focal lesions, 15 out of 53 (28.30%) and 15 out of 53 (28.30%), respectively.

**Table 4:** Diagnostic performance of conventional diagnostic assays and novel biomarker-based ELISAs for the detection of animals with focal lesions and any type of lesions.

METHOD	AUC	P value	CUT OFF	SE (%)	SP (%)	DV
	**DETECTION OF ANIMALS WITH FOCAL LESIONS**					
**ABCA13 ELISA**^a^	**0.837**	**<0.001**	**1.74**	**79.25**	**88.06**	**0.837**
MMP8 **ELISA**	0.771	<0.001	33.31	87.27	64.18	0.757
SPARC **ELISA**	0.802	<0.001	273.15	59.26	86.57	0.729
IDEXX ELISA^b^	0.541	0.434	28.39	14.55	100.00	0.573
IDEXX ELISA^c^			55.00	5.45	100.00	0.527
Fecal culture				5.45	100.00	0.527
Fecal PCR				9.09	100.00	0.545
Tissue culture				28.30	100.00*	0.642
Tissue PCR				28.30	100.00*	0.642
	**DETECTION OF ANIMALS WITH ANY LESION**					
**ABCA13 ELISA**^a^	**0.793**	**<0.001**	**1.39**	**69.41**	**86.57**	**0.780**
MMP8 **ELISA**	0.775	<0.001	33.17	87.50	64.18	0.758
SPARC **ELISA**	0.777	<0.001	291.55	51.72	88.06	0.699
IDEXX ELISA^b^	0.618	0.012	28.39	28.41	100.00	0.642
IDEXX ELISA^c^			55.00	21.59	100.00	0.608
Fecal culture				11.36	100.00	0.557
Fecal PCR				25.00	100.00	0.625
Tissue culture				38.37	100.00*	0.692
Tissue PCR				39.53	100.00*	0.698

AUC, area under the curve; P-value, it is the p-value of the AUC area, indicates whether the discrimination between animals with focal, multifocal, diffuse or any type of lesions and controls is significant; The cut-off point is expressed as ng/mL for the biomarkers and as a % of the positive reference sample for the IDEXX ELISA; SE, sensitivity; SP, specificity; DV, diagnostic value (semi-sum of the sensitivity and specificity); The control group consists of 67 animals, 6 animals with no lesions detected and 61 from a PTB-free farm; ABCA13, bovine ATP binding cassette subfamily A member 13; a, indicate that the number of animals with focal, multifocal, diffuse or with any type of lesions analysed for ABCA13 was 53, 17, 15 and 85, respectively; b, the cut off value used to estimate the sensitivity and specificity of the assay was calculated by ROC analysis; c, the cut off value used to estimate the sensitivity and specificity of the assay was the one established by the supplier

*, the estimation of the specificity for this methods is based on the analysis of 6 animals with no lesions, there is not availability of tissues for the 61 live animals from the PTB-free farm. The diagnostic methods with the best diagnostic value are shown in bold face.

Bovine biomarker-based ELISAs also showed a higher sensitivity and diagnostic value than the other diagnostic methods tested for overall detection of animals with any type of histological lesions (Table 4). The ABCA13-based ELISA was able to detect 59 out of 85 infected animals (69.41%) and as negative 58 out of 67 (86.57%) while the IDEXX ELISA was 100% specific but only detected as positive 19 out of 88 animals with lesions (21.59%) when the cut off was 55% or 25 out of 88 (28%) when the cut off used was 28.41%. Ante-mortem assays like fecal culture and PCR were 100% specific but only detected 10 out of 88 (11.36%) and 22 out of 88 (25%) animals with any type of lesions, respectively. Post-mortem assays like tissue culture and PCR were 100% specific and detected a higher percentage of animals with lesions, 33 out of 86 (38.37%) and 34 out of 86 (39.53%), respectively. All the tested methods had lower sensitivity than the biomarker-based ELISAs for the detection of animals with focal lesions and for the overall detection of animals with any type of histological lesion.

## 4. Discussion

The goals of PTB control programmes may vary from eradication in areas of low prevalence, control in areas with high prevalence or increased surveillance in an area with no prior history of disease [37]. Currently, the control of PTB at the herd level is based mainly on the identification and withdrawal of infected animals, especially MAP-shedding animals, to suppress sources of infection and maximize the productive life of the animals [45]. Therefore, the effectiveness of these control programs is strongly conditioned by the diagnostic methods used for the detection of infected animals. Host biomarkers may provide improved diagnostics for PTB increasing the effectiveness of control programmes.

To the best of our knowledge this is the first study where the detection of biomarkers in serum samples by specific ELISAs has been used and validated as a diagnostic tool for detection of MAP-infected animals. Our results indicate that the ABCA13, SPARC and MMP8-based ELISAs have higher AUC values and sensitivities than the IDEXX ELISA and other current diagnostic methods for detection of animals with focal, multifocal and any type of histopathological lesions, respectively. Specifically, the ABCA13-based ELISA had the best diagnostic performance for detection of animals with any type of lesions, it was able to detect 69.41% of these animals while the IDEXX ELISA, fecal culture and fecal PCR detected 28.41%, 11.36% and 25%, respectively. Therefore, the ABCA13-based ELISA greatly improves overall detection of animals with PTB-specific histological lesions. This is due to the fact that the biomarker-based ELISAs have better diagnostic accuracies for the focal and multifocal groups than the other diagnostic methods. In fact, the ABCA13-based ELISA showed the most accurate diagnostic performance for detection of animals with focal lesions. It had a 79.25% sensitivity vs. the 14.55%, 5.45% and 9.09% sensitivities for the IDEXX ELISA, fecal culture and fecal PCR, respectively. Likewise, the SPARC-based ELISA showed the best discriminatory power between the multifocal and control animals. It identified 66.67% of animals with multifocal lesions while the IDEXX ELISA, fecal culture and fecal PCR identified 27.78%, 16.67% and 33.33%, respectively. The MMP8-based ELISA showed the highest diagnostic accuracy for detection of animals with focal and multifocal lesions when they were grouped, it has a sensitivity of 96.97% vs. the 54.55%, 51.51% and 21.21% of the IDEXX ELISA, fecal culture and fecal PCR, respectively. These results indicate that the biomarker-based ELISAs consistently show higher sensitivity values than the current diagnostic methods.

The specificity of the IDEXX ELISA and the other conventional diagnostic methods (fecal and tissue culture and PCR) was higher (>97%) than that observed for the biomarker-based ELISAs in each histopathological group. The specificity values obtained for the best biomarker-based ELISAs in each histopathological group ranged from 58.21% of the MMP8-based ELISA for detection of the grouped multifocal and diffuse animals to 92.54% of the SPARC-based ELISA for detection of the multifocal group. Lower specificity values could be explained by the presence of infected animals that have not been recognized as such in the control group. This could be due to the low sensitivity of the diagnostic methods (IDEXX ELISA, and fecal culture and PCR) used to determine the PTB-status of the PTB-free farm [14]. There was a lack of control animals with no PTB-associated histopathological lesions detected. But even using histopathology to determine the PTB-status it is possible that some animals with focal lesions were treated as negative. The sections of intestine examined by histopathology correspond to the areas where lesions are most consistently found but the actual fraction of intestine analysed is very small which is not representative of what can be in the entire intestine. Another possible hypothesis to explain the lower specificity values obtained for the biomarker-based ELISAs is the presence in the PTB-free farm of animals infected by non-tuberculous mycobacteria that cause an increase in the levels of the selected biomarkers. However, this possibility needs to be explored before drawing any definitive conclusions. These considerations highlight the difficulty in establishing ideal control groups for the diagnosis of this disease.

Logistic regression analysis indicated that combination of biomarker-based ELISAs could improve the diagnostic performance (AUC values) of the ELISAs used individually for detection of some histopathological groups. On the whole, the diagnostic models had higher AUC values, increased sensitivities and decreased specificities. It will be necessary to assess whether the changes in sensitivity and specificity produced by the use of combined biomarkers are worthwhile as the use of such combined biomarkers would raise testing costs and complicate the diagnostic procedure. The sample size used in this study for validation of the biomarker-based ELISAs and the diagnostic models was moderate (n = 155) so further studies with a larger collection of samples need to be performed to validate that these biomarkers can be used to accurately diagnose all manifestations of MAP infection.

Up-regulation of these genes is a host response to infection of MAP. The ABCA13 gene is a member of the ABC gene subfamily A. ABC proteins facilitate translocation of heterogeneous substrates (lipids, peptides, proteins, ions, etc) across the cell membrane using energy acquired by the hydrolysis of ATP. Gene Ontology annotations related to this gene include ATPase and cholesterol transporter activity. In humans, the expression of ABCA13 is elevated in subjects with several pathologies as leukemia, prostate tumor, colorectal cancer, and tumor cell lines in central nervous system [46, 47]. Several studies have associated overexpression of ABCA13 with poor prognosis of cancer [48, 49]. ABCA13 is also considered a useful marker for predicting lymph node metastasis in resected gastric cancer patients in early stage [50]. Disruption to ABC transporter activity results in lipid accumulation and elevated levels of inflammatory cytokines in lung tissue [51]. Therefore, increased values of ABCA13 protein might be related to the chronic inflammation observed in the small intestine of MAP-infected animals.

There is evidence suggesting that MAP can induce the expression of matrix metallopeptidases (MMPs), which are the main proteases in the pathogenesis of mucosal ulcerations such inflammatory bowel disease [52]. Tissue inhibitors of MMP (TIMSPs) have been suggested as potential biomarkers for TB. TIMPs-1, -2 and -3 facilitate remodeling and repair of tissue following destruction by MMPs. For instance, the concentration of MMP-8 (or collagenase-2) has been demonstrated to decrease rapidly during TB treatment [53]. MMP9 and Timp1 are known to be up-regulated in tuberculosis infection and have been proposed as biomarkers for diagnosis of tuberculosis [54]. MMP8 is an MMP mainly produced by neutrophils and associated with many inflammatory conditions [55, 56]. Up-regulation of MMP-8 expression in peripheral blood of MAP infected animals may indicate that MMP-8 plays an important role in the inflammation and destruction of tissue observed in the development of PTB.

SPARC, also known as osteonectin, is a matrix protein that binds collagen, and is required for the development of granuloma-like structures during chronic infections [57]. In our previous RNA-Seq study [39], the protein-protein interaction analysis revealed a COL1A2 centered network containing a COLI1A2-SPARC functional interaction. SPARC and COL1A2 were upregulated in the peripheral blood gene expression profiles from the cows with diffuse lesions which suggests that the expression of both proteins could lead to a bad prognosis in MAP-infected cows. A SPARC-centered network was also expressed more strongly in *Mycobacterium bovis*-challenged monocyte-derived macrophages from bovine TB infected cows than in healthy cows [58].

Currently, it is impossible to identify all infected animals which makes it difficult to improve control and eradicate PTB. Even though, as we have confirmed here, the MAP specific antibody ELISA performs well for patent forms of infection, latent forms are mostly overlooked. For this reason, sensitive biomarker-based diagnostic assays that widen the detection range could be used: 1) in eradication campains by identifying cows before they commence fecal shedding of the pathogen; 2) to prevent the purchase of infected cattle that escape current detection methods avoiding rapid spread of PTB between herds; And 3) to reduce the potential of a herd to transmit infection to other domestic and wild ruminants. That is especially relevant since PTB is a disease that affects not only cattle but also other domestic (sheep, goats) [59] and wild ruminants (deer, fallow-deer) [60, 61], and other species like camelids [62], that have similar digestive characteristics although they are not true ruminants [63]. Improvement of the identification of MAP-infected animals through sensitive biomarker-based ELISAs could also help to study and confirm the potential role of MAP-infection as a common pathogenetic contributor to various autoimmune diseases [10].

In conclusion, biomarker-based ELISAs with good diagnostic performance could be used to improve PTB management. This is especially relevant for early detection of animals during latent stages of infection which are currently escaping detection. Individual or combined biomarker-based ELISAs could provide significant improvements in current PTB control programs when used together with traditional diagnostic methods.

## Supporting information

S1 Table (PDF)
