# Supplementary material for: Detection of latent forms of Mycobacterium avium subsp. paratuberculosis infection using host biomarker-based ELISAs greatly improves paratuberculosis diagnostic sensitivity
Source: PLoS One. 2020 Sep 3;15(9):e0236336. doi: 10.1371/journal.pone.0236336 (PMC7470414; doi:10.1371/journal.pone.0236336)
Supplement: S1 Table — (PDF) [file pone.0236336.s001.pdf]

ID	FARM	HISTOPATHOLOGICAL GROUP	ZN		INFECTION STATUS	ELISA FAM84A	ELISA DES	ELISA ABCA13	ELISA MMP8	ELISA SPARC	ELISA IDEXX	ELISA IDEXX (numerical value)	PCR FECES	PCR TISSUES	CULTURE FECES	CULTURE TISSUES	AGE (YEARS)	CLINICAL SIGNS
1	Noreña	FOCAL	POS	+	Infected	2,24	26,66	8,03	182,63	180,72	NEG	10,94	NEG	NEG	POS	NEG	6,58	UNKNOWN
2	Noreña	FOCAL	POS	+	Infected	1,71	38,25	3,85	111,72	1184,43	NEG	4,09	NEG	NEG	NEG	NEG	3,95	UNKNOWN
3	Noreña	FOCAL	POS	+	Infected	1,23	25,32	9,96	173,57	497,44	NEG	17,30	NEG	NEG	NEG	NEG	4,26	UNKNOWN
5	Noreña	FOCAL	NEG		Infected	2,16	43,96	7,42	995,85	2856,27	POS	157,76	NEG	NEG	NEG	POS	8,28	UNKNOWN
8	Noreña	FOCAL	POS	+	Infected	0,87	25,75	1,16	143,53	116,43	NEG	4,47	NEG	NEG	NEG	NEG	5,42	UNKNOWN
9	Noreña	FOCAL	NEG		Infected	0,26	11,98	0,75	124,83	109,87	NEG	1,35	NEG	NEG	NEG	NEG	2,5	UNKNOWN
10	Noreña	FOCAL	NEG		Infected	0,59	21,19	4,40	97,02	200,08	NEG	15,25	NEG	NEG	NEG	NEG	5,37	UNKNOWN
11	Noreña	FOCAL	NEG		Infected	0,51	32,43	11,09	17,04	97,22	NEG	10,61	NEG	NEG	NEG	NEG	8,71	UNKNOWN
12	Noreña	FOCAL	POS	+	Infected	0,74	11,31	1,91	52,65	135,68	NEG	9,32	NEG	NEG	NEG	NEG	2,61	UNKNOWN
14	Noreña	FOCAL	NEG		Infected	1,07	63,82	5,37	221,92	1254,76	NEG	16,16	NEG	NEG	NEG	NEG	5,42	UNKNOWN
15	Noreña	FOCAL	POS	+	Infected	0,29	54,77	2,31	127,11	15,43	NEG	33,24	NEG	NEG	NEG	NEG	5,93	UNKNOWN
16	Noreña	FOCAL	POS	+	Infected	1,11	51,22	3,36	121,03	2,71	NEG	9,97	NEG	NEG	NEG	NEG	6,14	UNKNOWN
17	Noreña	FOCAL	POS	+	Infected	0,34	41,82	9,53	36,94	1094,86	NEG	5,39	NEG	NEG	NEG	NEG	12,66	UNKNOWN
18	Noreña	FOCAL	NEG		Infected	0,19	42,11	5,67	41,50	238,17	NEG	9,86	NEG	NEG	NEG	NEG	3,67	UNKNOWN
19	Noreña	FOCAL	POS	+	Infected	0,61	43,73	3,49	518,01	881,14	NEG	6,47	NEG	NEG	NEG	NEG	6,79	UNKNOWN
20	Noreña	FOCAL	NEG		Infected	2,24	5,91	0,58	55,57	1258,85	POS	92,35	NEG	NEG	NEG	NEG	8,1	UNKNOWN
23	Noreña	FOCAL	NEG		Infected	0,20	22,74	4,06	39,47	659,23	NEG	28,39	NEG	NEG	NEG	NEG	7,75	UNKNOWN
24	Noreña	FOCAL	POS	+	Infected	0,23	16,50	3,07	79,78	1102,15	NEG	0,59	NEG	NEG	NEG	NEG	4,17	UNKNOWN
26	Noreña	FOCAL	NEG		Infected	0,55	57,67	3,90	109,36	13,19	NEG	6,79	NEG	POS	NEG	POS	4,08	UNKNOWN
28	Noreña	FOCAL	POS	+	Infected	1,57	37,21	10,38	209,50	1042,66	NEG	3,39	NEG	POS	NEG	NEG	7	UNKNOWN
29	Noreña	FOCAL	POS	+	Infected	1,10	52,38	2,84	151,29	322,52	NEG	3,07	NEG	POS	NEG	NEG	8,44	UNKNOWN
31	Noreña	FOCAL	POS	+	Infected	0,25	9,27	0,52	43,27	1325,44	NEG	11,80	NEG	NEG	NEG	NEG	5,42	UNKNOWN
32	Noreña	FOCAL	POS	+	Infected	0,44	11,35	3,26	59,87	1005,29	NEG	4,15	POS	POS	NEG	POS	6,25	UNKNOWN
33	Noreña	FOCAL	POS	+	Infected	-0,03	8,49	0,66	281,24	1608,38	NEG	1,24	NEG	NEG	NEG	NEG	5,15	UNKNOWN
34	Noreña	FOCAL	POS	+	Infected	0,13	15,92	0,64	80,28	980,47	NEG	2,75	POS	NEG	POS	NEG	4,5	UNKNOWN
35	Noreña	FOCAL	POS	+	Infected	0,13	12,43	2,51	155,50	1320,81	NEG	10,02	NEG	NEG	NEG	NEG	4,29	UNKNOWN
36	Noreña	FOCAL	POS	+	Infected	0,90	16,15	4,47	319,77	460,41	NEG	7,92	NEG	POS	NEG	POS	7,36	UNKNOWN
1	SERIDA	FOCAL	POS	+	Infected	1,36	46,06	0,92	38,08	117,87	NEG	47,25	NEG	NEG	NEG	NEG	4,1	NO
2	SERIDA	FOCAL	POS	+	Infected	0,23	56,24	3,13	156,08	273,15	NEG	3,57	NEG	NEG	NEG	NEG	5,04	NO
1	Soto	FOCAL	NEG		Infected	0,98	24,24	5,25	338,27	212,85	NEG	3,27	NEG	NEG	NEG	NEG	8,21	NO
7	Soto	FOCAL	POS	+	Infected	1,86	14,78		17,62	1071,64	NEG	0,61	NEG	UNDETERMINED	NEG	UNDETERMINED	6,95	NO
8	Soto	FOCAL	POS	+	Infected	0,69	20,95	5,60	12,98	507,42	NEG	2,65	NEG	POS	NEG	POS	4,71	NO
13	Soto	FOCAL	NEG		Infected	3,00	14,06	5,77	88,27	190,47	NEG	2,33	NEG	NEG	NEG	NEG	5,69	NO
17	Soto	FOCAL	NEG		Infected	1,49	24,08	21,96	33,64	121,31	NEG	5,27	NEG	NEG	NEG	NEG	5,38	NO
18	soto	FOCAL	NEG		Infected	1,04	34,60	18,36	33,31	156,97	NEG	1,71	NEG	NEG	NEG	NEG	6,94	NO
19	Soto	FOCAL	NEG		Infected	3,93	71,82	22,77	177,88	1152,72	NEG	9,17	NEG	POS	NEG	NEG	6,19	NO
25	Soto	FOCAL	NEG		Infected	0,40	5,23	0,79	56,96	6381,00	NEG	1,40	NEG	UNDETERMINED	NEG	UNDETERMINED	5,99	NO
28	Soto	FOCAL	NEG		Infected	7,70	53,90	12,58	196,39	828,81	NEG	0,81	POS	POS	NEG	POS	8,39	NO
31	Soto	FOCAL	NEG		Infected	0,16	78,97	7,04	18,05	868,24	NEG	5,51	NEG	POS	NEG	NEG	5,33	NO
36	Soto	FOCAL	POS	+	Infected	0,25	26,14	1,90	89,28	32,04	NEG	11,72	NEG	NEG	NEG	NEG	8,17	NO
38	Soto	FOCAL	NEG		Infected	2,11	16,75	7,04	540,06	1095,48	NEG	28,63	NEG	NEG	NEG	NEG	8,89	NO
41	Soto	FOCAL	NEG		Infected	1,61	29,48	14,98	75,85	245,65	NEG	1,03	NEG	POS	NEG	POS	7,15	NO
43	Soto	FOCAL	NEG		Infected	0,77	37,90	16,32	55,82	315,21	NEG	3,50	NEG	NEG	NEG	NEG	4,14	NO
44	Soto	FOCAL	NEG		Infected	3,17	24,70	1,07	41,25	382,45	NEG	6,20	NEG	NEG	NEG	POS	5,45	NO
51	Soto	FOCAL	NEG		Infected	0,44	13,40		13,88	292,29	NEG	2,79	NEG	POS	NEG	NEG	9,48	NO
52	Soto	FOCAL	NEG		Infected	1,92	30,13	22,55	192,46	825,13	POS	133,17	POS	POS	NEG	POS	6,59	NO
53	Soto	FOCAL	NEG		Infected	0,42	10,82	3,82	214,57	3823,02	NEG	0,74	NEG	POS	NEG	NEG	6,82	NO
70	Soto	FOCAL	POS	+	Infected	0,45	23,10	8,06	23,76	645,92	NEG	3,83	NEG	NEG	NEG	NEG	4,35	NO
78	Soto	FOCAL	POS	+	Infected	0,38	26,54	0,70	90,93	291,55	NEG	1,09	NEG	NEG	NEG	POS	4,39	NO
106	Soto	FOCAL	NEG		Infected	0,08	12,91	52,64	118,94		NEG	2,71	NEG	POS	NEG	POS	2,72	NO
112	Soto	FOCAL	NEG		Infected	0,18	31,20	5,64	93,34	153,43	NEG	37,16	NEG	NEG	NEG	POS	2,97	NO
114	Soto	FOCAL	NEG		Infected	0,38	78,39	24,21	43,91	189,02	NEG	3,77	POS	POS	POS	POS	2,6	NO
118	Soto	FOCAL	POS	+	Infected	0,37	24,38	0,50	16,28	174,60	NEG	1,29	NEG	NEG	NEG	NEG	3,74	NO
143	Soto	FOCAL	NEG		Infected	0,72	37,25	4,88	58,48	1,76	NEG	2,42	NEG	NEG	NEG	POS	1,26	NO
160	Soto	FOCAL	POS	+	Infected	6,60	13,26	1,74	37,57	254,13	NEG	2,37	NEG	NEG	NEG	POS	2,12	NO
6	Noreña	MULTIFOCAL	POS	+	Infected	0,41	18,59	1,39	51,23	942,04	NEG	6,57	NEG	NEG	NEG	POS	6,58	UNKNOWN
22	Noreña	MULTIFOCAL	POS	+	Infected	8,91	18,27	1,76	160,32	18005,52	POS	68,86	NEG	NEG	NEG	NEG	5,34	UNKNOWN
27	Noreña	MULTIFOCAL	POS	+	Infected	3,04	35,52	3,69	118,76	513,94	POS	148,65	POS	POS	NEG	NEG	5,65	UNKNOWN
30	Noreña	MULTIFOCAL	POS	+	Infected	0,87	24,81	1,65	204,43	1267,13	NEG	3,34	NEG	NEG	NEG	NEG	5,58	UNKNOWN

ID	FARM	HISTOPATHOLOGICAL GROUP	ZN		INFECTION STATUS	ELISA FAM84A	ELISA DES	ELISA ABCA13	ELISA MMP8	ELISA SPARC	ELISA IDEXX	ELISA IDEXX (numerical value)	PCR FECES	PCR TISSUES	CULTURE FECES	CULTURE TISSUES	AGE (YEARS)	CLINICAL SIGNS
6	Soto	MULTIFOCAL	POS	+	Infected	0,244	61,382	1,09	94,86	109,84	NEG	3,239	NEG	NEG	NEG	NEG	6,63	NO
11	Soto	MULTIFOCAL	POS	+	Infected	0,40	11,62	0,56	59,87	948,75	NEG	1,50	NEG	NEG	NEG	POS	7,31	NO
46	Soto	MULTIFOCAL	POS	+	Infected	0,71	4,60	1,17	70,86	200,58	NEG	2,98	NEG	NEG	NEG	NEG	2,75	SI
47	Soto	MULTIFOCAL	POS	++	Infected	0,14	4,47	0,97	33,17	252,41	POS	67,58	POS	POS	POS	POS	5,51	NO
48	Soto	MULTIFOCAL	POS	+	Infected	1,39	36,80	2,33	28,35	892,33	NEG	4,82	NEG	NEG	NEG	POS	9,67	NO
54	Soto	MULTIFOCAL	POS	+	Infected	1,186	52,404	0,6	53,92	1163,56	NEG	12,56	POS	POS	POS	NEG	6,14	NO
63	Soto	MULTIFOCAL	POS	+	Infected	1,25	25,50	2,26	243,72	50,40	NEG	7,60	NEG	NEG	NEG	NEG	6,42	SI
64	Soto	MULTIFOCAL	POS	+	Infected	1,65	43,61	0,37	50,33	354,94	POS	186,89	POS	POS	POS	POS	3,62	NO
65	Soto	MULTIFOCAL	POS	+	Infected	0,10	10,38	0,45	29,59	1351,29	NEG	3,46	NEG	POS	NEG	NEG	7,7	NO
76	Soto	MULTIFOCAL	POS	+	Infected	0,87	16,83		18,17	1724,50	NEG	4,86	NEG	POS	NEG	NEG	5,72	NO
93	Soto	MULTIFOCAL	POS	+	Infected	1,09	37,36	1,09	26,92	1234,00	NEG	2,12	NEG	NEG	NEG	NEG	4,76	NO
97	Soto	MULTIFOCAL	POS	++	Infected	0,07	33,51	6	42,48	165,74	POS	131,05	POS	POS	NEG	POS	2,96	SI
110	Soto	MULTIFOCAL	NEG		Infected	0,48	23,93	4,37	53,25	524,74	NEG	2,96	NEG	POS	NEG	POS	4,03	NO
146	Soto	MULTIFOCAL	POS	+	Infected	0,265	28,804	0,70	39,47	116,10	NEG	3,407	POS	NEG	NEG	POS	2,57	NO
25	Noreña	DIFUSSE INTERMEDIATE	POS	++	Infected	2,70	22,88	1,41	266,79	175,69	POS	137,07	POS	POS	NEG	POS	8,44	UNKNOWN
3	SERIDA	DIFUSSE INTERMEDIATE	POS	++	Infected	3,27	59,25	1,22	224,78	196,55	NEG	4,58	NEG	NEG	NEG	NEG	4,85	NO
4	SERIDA	DIFUSSE INTERMEDIATE	POS	++	Infected	1,17	73,31	3,59	59,37	216,11	NEG	41,55	NEG	NEG	NEG	NEG	2,15	NO
5	Soto	DIFUSSE INTERMEDIATE	POS	++	Infected	1,06	23,85	1,20	98,90	40,61	POS	205,94	POS	POS	NEG	POS	5,22	SI
26	Soto	DIFUSSE INTERMEDIATE	POS	+	Infected	0,28	5,25	6,89	73,10	903,81	POS	187,69	POS	POS	NEG	POS	5,46	NO
27	Soto	DIFUSSE PAUCIBACILLARY	POS	+	Infected	1,79	6,37	2,75	60,20	92,95	POS	283,13	POS	POS	NEG	POS	3,44	NO
30	Soto	DIFUSSE INTERMEDIATE	POS	+	Infected	0,49	70,83	10,02	42,59	174,99	NEG	2,71	NEG	NEG	NEG	NEG	4,14	SI
32	Soto	DIFUSSE INTERMEDIATE	POS	+	Infected	2,55	8,29	2,53	293,91	359,49	POS	241,18	POS	POS	POS	POS	4,47	SI
49	Soto	DIFUSSE MULTIBACILAR	POS	+	Infected	2,47	36,59	4,06	85,33	98,97	POS	94,49	POS	POS	POS	POS	4,45	SI
59	Soto	DIFUSSE INTERMEDIATE	POS	++	Infected	1,50	50,79	5,74	716,50	7,73	POS	288,75	POS	POS	NEG	POS	7,02	SI
68	Soto	DIFUSSE INTERMEDIATE	POS	++	Infected	2,33	5,70	2,56	63,79	70,75	POS	254,72	POS	POS	POS	POS	6,01	SI
87	Soto	DIFUSSE INTERMEDIATE	POS	++	Infected	0,21	66,80	1.29	96,93	156,32	POS	113,49	POS	POS	NEG	NEG	5,09	SI
88	Soto	DIFUSSE MULTIBACILAR	POS	+++	Infected	1,12	15,58	1,26	116,06	124,70	POS	286,51	POS	POS	NEG	POS	2,92	SI
99	Soto	DIFUSSE MULTIBACILAR	POS	+++	Infected	0,34	60,20	0,58	66,60	112,74	POS	157,83	POS	POS	POS	POS	4,82	SI
122	Soto	DIFUSSE INTERMEDIATE	POS	++	Infected	0,10	29,54	0,63	40,23	203,77	NEG	19,60	NEG	NEG	NEG	NEG	6,19	NO
12	Soto	NO LESIONS	NEG		Not infected	0,22	7,68	5,98	17,69	145,08	NEG	1,72	NEG	NEG	NEG	NEG	3,58	NO
80	Soto	NO LESIONS	NEG		Not infected	1,12	55,68	9,58	111,24	88,75	NEG	8,54	NEG	NEG	NEG	NEG	2,86	NO
94	Soto	NO LESIONS	NEG		Not infected	2,34	24,62	10,65	64,13	114,32	NEG	1,26	NEG	NEG	NEG	NEG	2,7	NO
113	Soto	NO LESIONS	NEG		Not infected	0,32	5,99	0,43	97,00	8,98	NEG	2,45	NEG	NEG	NEG	NEG	1,27	NO
4	Noreña	NO LESIONS	NEG		Not infected	3,12	21,04	2,09	242,20	1477,86	NEG	5,44	NEG	NEG	NEG	NEG	3,26	UNKNOWN
13	Noreña	NO LESIONS	NEG		Not infected	9,49	21,18	1,34	158,69	661,62	NEG	8,84	NEG	NEG	NEG	NEG	0,81	UNKNOWN
1	Flor	PTB-FREE FARM			Not infected	0,08	11,66	0,57	67,38	216,31	NEG	3,23	NEG		NEG		4,50	NO
2	Flor	PTB-FREE FARM			Not infected	0,54	27,42	1,47	499,75	219,25	NEG	9,22	NEG		NEG		7,58	NO
3	Flor	PTB-FREE FARM			Not infected	0,96	14,78	0,86	12,96	228,19	NEG	1,32	NEG		NEG		3,25	NO
4	Flor	PTB-FREE FARM			Not infected	1,20	29,50	1,96	12,23	165,46	NEG	5,47	NEG		NEG		3,50	NO
5	Flor	PTB-FREE FARM			Not infected	0,80	24,68	0,79	16,02	174,64	NEG	1,84	NEG		NEG		3,83	NO
6	Flor	PTB-FREE FARM			Not infected	1,03	17,43	0,79	16,75	374,14	NEG	0,56	NEG		NEG		3,67	NO
7	Flor	PTB-FREE FARM			Not infected	0,26	24,22	0,83	15,53	156,84	NEG	4,35	NEG		NEG		4,25	NO
8	Flor	PTB-FREE FARM			Not infected	0,46	20,37	0,93	19,10	260,91	NEG	4,35	NEG		NEG		3,92	NO
9	Flor	PTB-FREE FARM			Not infected	0,83	20,14	0,71	38,69	260,13	NEG	11,14	NEG		NEG		10,08	NO
10	Flor	PTB-FREE FARM			Not infected	6,14	25,19	0,92	27,38	180,86	NEG	3,31	NEG		NEG		9,75	NO
11	Flor	PTB-FREE FARM			Not infected	0,19	17,18	0,69	188,34	227,51	NEG	0,20	NEG		NEG		3,92	NO
12	Flor	PTB-FREE FARM			Not infected	0,70	9,83	0,56	78,43	222,34	NEG	3,95	NEG		NEG		5,75	NO
13	Flor	PTB-FREE FARM			Not infected	0,38	8,63	0,51	50,95	327,47	NEG	20,28	NEG		NEG		5,67	NO
14	Flor	PTB-FREE FARM			Not infected	0,22	17,42	0,58	10,89	132,46	NEG	4,31	NEG		NEG		3,25	NO
15	Flor	PTB-FREE FARM			Not infected	0,30	25,33	0,83	36,90	295,09	NEG	9,70	NEG		NEG		4,67	NO
16	Flor	PTB-FREE FARM			Not infected	0,36	13,31	0,57	9,91	107,56	NEG	2,79	NEG		NEG		3,83	NO
17	Flor	PTB-FREE FARM			Not infected	0,40	58,42	1,28	17,36	153,81	NEG	13,85	NEG		NEG		4,83	NO
18	Flor	PTB-FREE FARM			Not infected	0,25	18,41	0,58	25,67	342,66	NEG	2,79	NEG		NEG		6,42	NO
19	Flor	PTB-FREE FARM			Not infected	1,82	18,66	0,59	12,84	401,25	NEG	3,31	NEG		NEG		3,25	NO
20	Flor	PTB-FREE FARM			Not infected	0,37	9,60	0,62	47,16	149,61	NEG	7,27	NEG		NEG		3,58	NO
21	Flor	PTB-FREE FARM			Not infected	1,01	12,65	0,88	29,82	126,38	NEG	12,65	NEG		NEG		4,67	NO
22	Flor	PTB-FREE FARM			Not infected	0,47	31,14	0,86	10,64	149,61	NEG	4,43	NEG		NEG		7,00	NO
23	Flor	PTB-FREE FARM			Not infected	0,20	10,40	0,54	12,35	145,89	NEG	16,41	NEG		NEG		9,08	NO

ID	FARM	HISTOPATHOLOGICAL GROUP	ZN	INFECTION STATUS	ELISA FAM84A	ELISA DES	ELISA ABCA13	ELISA MMP8	ELISA SPARC	ELISA IDEXX	ELISA IDEXX (numerical value)	PCR FECES	PCR TISSUES	CULTURE FECES	CULTURE TISSUES	AGE (YEARS)	CLINICAL SIGNS
24	Flor	PTB-FREE FARM		Not infected	2,44	22,90	0,81	39,95	736,30	NEG	6,87	NEG		NEG		4,50	NO
25	Flor	PTB-FREE FARM		Not infected	0,28	17,46	0,68	16,38	101,08	NEG	20,76	NEG		NEG		4,50	NO
26	Flor	PTB-FREE FARM		Not infected	0,18	30,27	0,56	30,89	252,21	NEG	10,14	NEG		NEG		4,33	NO
27	Flor	PTB-FREE FARM		Not infected	7,64	22,76	0,74	38,73	248,64	NEG	-0,32	NEG		NEG		4,17	NO
28	Flor	PTB-FREE FARM		Not infected	1,12	15,21	1,11	110,91	239,22	NEG	14,89	NEG		NEG		3,08	NO
29	Flor	PTB-FREE FARM		Not infected	0,86	19,47	0,66	11,13	114,46	NEG	1,12	NEG		NEG		9,86	NO
30	Flor	PTB-FREE FARM		Not infected	0,78	18,70	0,76	495,65	3,38	NEG	9,78	NEG		NEG		2,58	NO
31	Flor	PTB-FREE FARM		Not infected	2,12	38,75	1,22	143,03	32,87	NEG	10,34	NEG		NEG		2,25	NO
32	Flor	PTB-FREE FARM		Not infected	0,33	45,94	1,17	68,04	1,33	NEG	7,62	NEG		NEG		2,83	NO
33	Flor	PTB-FREE FARM		Not infected	0,26	17,32	0,59	14,06	3,67	NEG	13,13	NEG		NEG		5,25	NO
34	Flor	PTB-FREE FARM		Not infected	0,72	52,39	0,89	11,01	12,76	NEG	2,55	NEG		NEG		2,33	NO
35	Flor	PTB-FREE FARM		Not infected	2,94	42,54	0,80	89,91	1,14	NEG	4,87	NEG		NEG		2,50	NO
36	Flor	PTB-FREE FARM		Not infected	0,26	34,05	0,83	25,42	14,69	NEG	5,95	NEG		NEG		2,25	NO
37	Flor	PTB-FREE FARM		Not infected	0,27	13,97	0,54	11,25	290,29	NEG	2,12	NEG		NEG		2,25	NO
38	Flor	PTB-FREE FARM		Not infected	0,29	46,12	1,11	13,82	2,56	NEG	3,67	NEG		NEG		4,50	NO
39	Flor	PTB-FREE FARM		Not infected	1,30	21,33	0,54	17,73	3,35	NEG	4,39	NEG		NEG		2,58	NO
40	Flor	PTB-FREE FARM		Not infected	0,22	33,77	0,62	9,91	0,97	NEG	12,77	NEG		NEG		2,50	NO
41	Flor	PTB-FREE FARM		Not infected	0,77	22,12	0,51	14,31	231,31	NEG	7,19	NEG		NEG		6,92	NO
42	Flor	PTB-FREE FARM		Not infected	0,75	25,61	2,20	22,00	215,45	NEG	5,67	NEG		NEG		7,25	NO
43	Flor	PTB-FREE FARM		Not infected	2,58	14,96	0,93	11,86	5,09	NEG	7,54	NEG		NEG		1,75	NO
44	Flor	PTB-FREE FARM		Not infected	3,53	12,74	0,67	78,10	108,23	NEG	0,12	NEG		NEG		0,67	NO
45	Flor	PTB-FREE FARM		Not infected	6,42	15,70	0,97	12,91	0,94	NEG	0,18	NEG		NEG		1,25	NO
46	Flor	PTB-FREE FARM		Not infected	0,51	9,92	0,69	22,05	1,10	NEG	5,97	NEG		NEG		1,42	NO
47	Flor	PTB-FREE FARM		Not infected	1,04	14,86	0,88	21,77	0,87	NEG	4,94	NEG		NEG		1,92	NO
48	Flor	PTB-FREE FARM		Not infected	0,59	13,23	0,53	19,83	0,88	NEG	5,73	NEG		NEG		1,50	NO
49	Flor	PTB-FREE FARM		Not infected	0,48	18,66	0,72	188,69	74,60	NEG	1,15	NEG		NEG		1,75	NO
50	Flor	PTB-FREE FARM		Not infected	0,72	17,61	0,90	27,58	77,71	NEG	3,32	NEG		NEG		1,83	NO
51	Flor	PTB-FREE FARM		Not infected	0,51	18,87	1,10	18,31	45,13	NEG	6,81	NEG		NEG		0,50	NO
52	Flor	PTB-FREE FARM		Not infected	0,21	21,91	1,28	17,89	0,89	NEG	19,11	NEG		NEG		0,83	NO
53	Flor	PTB-FREE FARM		Not infected	8,84	18,09	1,24	36,99	0,90	NEG	1,45	NEG		NEG		1,33	NO
54	Flor	PTB-FREE FARM		Not infected	0,98	19,18	1,14	16,65	1,30	NEG	1,69	NEG		NEG		1,50	NO
55	Flor	PTB-FREE FARM		Not infected	2,07	10,52	0,68	19,28	1,09	NEG	0,30	NEG		NEG		1,83	NO
56	Flor	PTB-FREE FARM		Not infected	1,09	7,79	0,47	25,78	1,68	NEG	0,60	NEG		NEG		0,67	NO
57	Flor	PTB-FREE FARM		Not infected	0,32	18,41	1,00	19,14	0,91	NEG	1,51	NEG		NEG		1,33	NO
58	Flor	PTB-FREE FARM		Not infected	0,20	68,99	3,52	22,74	0,85	NEG	1,57	NEG		NEG		1,08	NO
59	Flor	PTB-FREE FARM		Not infected	0,38	13,37	1,26	16,79	0,93	NEG	3,98	NEG		NEG		0,83	NO
60	Flor	PTB-FREE FARM		Not infected	0,34	13,52	1,10	13,88	0,89	NEG	14,41	NEG		NEG		1,25	NO
61	Flor	PTB-FREE FARM		Not infected	0,30	35,13	3,28	400,33	0,90	NEG	11,28	NEG		NEG		1,00	NO
